# Microbubble Formation and Hemolysis in Pulsed Field Ablation for Treating Arrhythmia: Risks, Mechanisms, and Prevention

**DOI:** 10.1111/pace.70035

**Published:** 2025-12-05

**Authors:** Pegah Ranjbartehrani

**Affiliations:** ^1^ Synaptic Medical Carlsbad California USA

**Keywords:** arrythmia, cardiac ablation, hemolysis, microbubbles, pulsed field ablation

## Abstract

Pulsed Field Ablation (PFA) is an emerging energy modality for cardiac ablation, offering shorter atrial dwell times and reduced collateral damage compared to thermal methods. However, energy‐related complications, specifically gaseous microbubble formation and hemolysis, require further evaluation. Microbubble formation, driven by electrolysis, boiling, and degassing, poses a potential risk for embolic events. Similarly, hemolysis results from unintended energy dissipation into the bloodstream, affecting kidney function. Both complications can be mitigated through optimized waveform and catheter design and other procedural strategies. Enhancing PFA safety requires a deeper understanding of its biophysical interactions and continuous refinement of ablation protocols to minimize risks while preserving clinical efficacy.

**Abbreviations**
AKI: acute kidney injury
DWI: diffusion‐weighted imaging
FLAIR: fluid‐attenuated inversion recovery
IFU: instructions for use
MRI: magnetic resonance imaging
PFA: pulsed field ablation
PFH: plasma free hemoglobin
RBC: red blood cell
SCE: silent cerebral events
SCL: silent cerebral lesions
TIA: transient ischemic attack

## 1 Introduction

Pulsed Field Ablation (PFA) has gained increasing attention as a promising energy modality for cardiac ablation. While PFA demonstrates similar efficacy to thermal ablation, it is favored for its shorter atrial dwell time and lower risk of life‐threatening collateral damage compared to traditional thermal methods [1]. Unlike radiofrequency and cryoablation, which rely on extreme temperatures to destroy cardiac tissue, PFA uses high‐voltage, short, electrical pulses to create microscopic pores in cell membranes, disrupting cellular homeostasis and leading to cell death. However, despite its growing adoption and generally favorable safety profile in clinical trials and post‐market studies, safety concerns remain and warrant ongoing evaluation.

Although several general safety concerns have been reported in clinical data on PFA, this review specifically focuses on two of the emerging energy‐related complications that arise due to the direct effects of the applied electrical field: gaseous microbubble formation and hemolysis. These complications stem from the unique biophysical interactions between pulsed electric fields and biological tissues, distinguishing them from procedural or mechanical risks. Understanding their mechanisms, clinical implications, and mitigation strategies is essential for ensuring the continued safe use of PFA in clinical practice.

## 2 Gaseous Microbubble Formation

Gaseous microbubble formation during the application of PFA for cardiac ablation is a critical concern due to its associated risk of gaseous microemboli, which can potentially lead to silent cerebral lesions (SCL), and stroke [2, 3, 4, 5]. Addressing this complication requires a thorough understanding of the mechanisms of bubble formation, the contributing factors, and its clinical implications, combined with the development and implementation of effective preventive strategies.

### 2.1 Mechanisms of Bubble Formation

Microbubbles are small gas‐filled pockets formed during PFA in liquid media such as blood. Their formation can be attributed to three main mechanisms [6]:

1. Electrochemical Reactions: At the electrode interface, electrolysis and ion oxidation generate gases that form microbubbles.
2. Boiling and Thermal Effects: Localized boiling can occur during high‐energy pulsing, though PFA's minimal thermal effects generally limit this risk compared to thermal ablation techniques.
3. Degassing: Dissolved gases are released due to changes in pressure, temperature, or energy input.

Bubble formation often begins with electrolysis and is exacerbated by high‐pressure shock waves that cause rapid gas expansion. These shock waves cause extrusion of dissolved air from the blood, while distortions in voltage waveforms, signaling the initiation of arcing, further amplify gas release and bubble growth [7].

### 2.2 Preclinical Insights Into Factors Influencing Microbubble Generation

Studies have shown that cathodal pulses generate significantly more bubbles than anodal pulses due to the higher volume of hydrogen gas released. For instance, in a porcine model, cathodal pulses produced 124‐fold more gas at 200 J energy levels compared to anodal pulses (85.4 vs. 0.61 µL) [8]. The gas volume scales linearly with delivered charge, underscoring the importance of minimizing energy settings to reduce bubble generation [5]. Additionally, microbubbles were observed to adhere to the electrode surface for several seconds, reducing effective electrode surface area and lowering arcing thresholds during subsequent pulses [5]. Moreover, the bubbles that attach to the surface of the electrodes for longer times, have more potential to grow into larger bubbles [6].

Several studies reported that monophasic pulse protocols are associated with larger gas volumes compared to biphasic pulses due to continuous accumulation of electrochemical byproducts. However, biphasic pulses alternate polarity, reversing electrode polarization effects and limiting gas formation due to electrolysis [2, 6, 9, 10]. The formation of microbubbles is also strongly influenced by the frequency of the applied pulses [6]. High‐frequency biphasic pulses significantly reduce electrolysis‐related bubble formation by limiting faradaic reactions [6, 11].

### 2.3 Clinical Implications

The clinical implications of microbubbles depend on their size and persistence. Small bubbles (<38 µm in diameter) dissolve rapidly within 6–10 s and are unlikely to cause ischemic complications [12, 13]. However, larger bubbles, particularly those formed during high‐energy monophasic pulses (100–200 J), may impair blood flow transiently and have been associated with ST‐segment elevations and punctate ischemic lesions observed in postprocedural imaging [2, 14].

Several clinical studies have examined the incidence of silent cerebral lesions (SCLs) and stroke following cardiac ablation with PFA. However, it cannot be conclusively stated that the observed rates of SCLs and stroke are solely energy‐related and only attributable to PFA itself. Procedural factors, such as catheter exchanges and other mechanisms by which bubbles may enter the bloodstream, likely contribute to these outcomes. The Table 1 summarizes the rates of silent cerebral lesions (SCL), events (SCE), and strokes, reported in clinical studies involving PFA for cardiac ablation.

**TABLE 1:** Clinical studies investigating silent cerebral events.

Study name/Author	SCL/SCE %	Stroke/TIA %
Reddy et al. (2021) (IMPULSE, PEFCAT, PEFCAT II) [15]	NA	0.8% TIA
Schmidt et al. (2022) (5S) [16]	19% silent cerebral injury	1.05% minor strokes
(MANIFEST‐17K) [17]	9.4% asymptomatic MRI abnormalities	0.12% stroke
Patel et al. (2024) (ADVENT‐Neurological) [18]	17.65% SCE/SCL positive	1 patient had TIA, 1 stroke (not in the neurological subgroup)
Verma et al. (2023) (PULSED‐AF) [19]	8.9% SCL	0% TIA
Sanders et al. (2024) (VOLT) [20]	9.4% SCL	0% strokes
Anic et al. (2023) (CENTAURI) [21]	11.4% SCE (DWI), 0% SCL (FLAIR)	1.2% stroke, 0% TIA
Reddy et al. (2024) (Sphere‐360) [22]	10% (FLAIR‐positive), 8% (FLAIR‐negative)	0% stroke/TIA
Reddy et al. (2023) (Sphere‐9) [23]	7.9% SCE (DWI‐positive/FLAIR‐negative), 6.7% SCL (DWI‐positive/FLAIR‐positive)	0% stroke/TIA
Duytschaever et al. (2024) (SmartfIRE) [24]	6.7% microemboli lesions (resolved at follow‐up), 3.3% SCL	0.7% stroke, 0%TIA
Reinsch et al. (2022) [25]	3% SCL (resolved at follow‐up)	0% stroke
Turagam et al. (2023) (PULSE‐EU) [26]	18.7% (DWI positive/FLAIR‐negative)	0% stroke

### 2.4 Preventive Strategies

**FIGURE 1:**
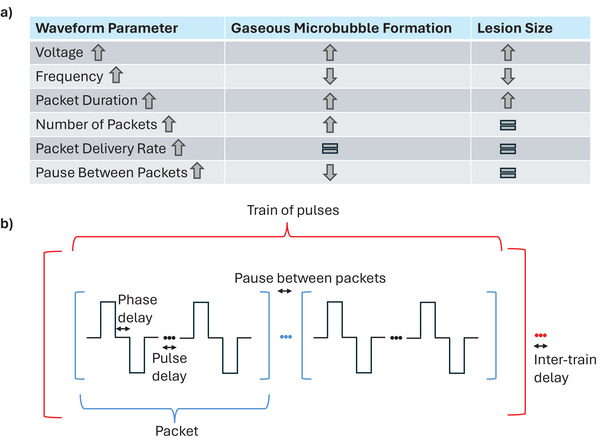
(a) Effects of waveform parameters on lesion size and gaseous microbubble formation. (b) Illustrations of a basic biphasic PFA waveform structure. [Colour figure can be viewed at wileyonlinelibrary.com]

To minimize microbubble formation and reduce the associated risks of silent cerebral lesions and stroke, we can implement the following strategies:

1. Better Electrode Design: Avoiding sharp edges and regions of high current density to reduce thermal hotspots and arcing.
2. Waveform and Energy Optimization: Waveforms must be carefully adjusted to achieve effective tissue ablation while minimizing microbubble production. Biphasic and high‐frequency waveforms have been shown to generate fewer microbubbles compared to monophasic waveforms. Additionally, optimizing duty cycles and delivered energy, along with incorporating pauses between pulses, allows for adequate gas and heat dissipation and further reduces bubble formation (Figure 1).

## 3 Hemolysis During Cardiac Ablation With Pulsed Field Ablation

Hemolysis is an energy‐related complication of pulsed field ablation (PFA) for cardiac ablation that gained attention after reports of acute kidney injury (AKI) in two patients undergoing PFA for arrhythmia [27]. Clinical studies have associated hemolysis with complications such as hemoglobinuria and, in rare cases, AKI [17, 28, 29, 30]. Intravascular hemolysis leads to AKI only when it reaches a severe threshold. Currently, no clinical data establishes a quantitative relationship between hemolysis severity and the onset or progression of AKI.

Full electrode‐tissue contact, minimizing direct blood exposure, is crucial in preventing hemolysis. Consequently, catheter design, including electrode size, spacing, configuration, and the avoidance of high current density, plays a significant role. Additionally, blood flow velocity during ablation, influenced by hemorheologic factors and anticoagulation, may also contribute to hemolysis development.

Effectively addressing this complication requires a comprehensive understanding of the mechanisms behind hemolysis and its clinical implications, along with the development and implementation of targeted preventive strategies.

### 3.1 Mechanisms of Hemolysis and Acute Kidney Injury After Pulsed Field Ablation

Hemolysis occurs when electrical currents from the electrodes inadvertently enter the bloodstream instead of targeting tissue, leading to damage to blood cells. The higher electrical conductivity of blood compared to cardiac tissues, further increases the likelihood of PFA energy being transmitted into the blood, especially in cases of direct electrode contact with blood [2, 31]. This process causes break down of the red blood cells (RBCs), releasing free hemoglobin, heme, and iron. These byproducts can provoke inflammation, immune responses, and endothelial dysfunction. While protective mechanisms involving haptoglobin, hemopexin, and apotransferrin help scavenge these molecules, excessive hemolysis, caused by PFA energy, overwhelms these systems, leading to systemic effects. The kidneys, responsible for clearing free hemoglobin, are especially vulnerable when these natural defenses fail [32].

### 3.2 Preclinical Insights Into Factors Influencing Hemolysis

Preclinical studies have shown that hemolysis during pulsed field ablation (PFA) for treating arrhythmia is influenced by electric field intensity, number of lesions, and catheter‐tissue contact.

Fiserova et al. [33] conducted an in vitro study using human blood samples exposed to varying electric field strengths (250–1500 V/cm) delivered as short, high‐intensity bipolar pulses. Hemolysis was assessed by measuring cell‐free hemoglobin levels. Significant hemolysis was observed at field strengths of 1000 V/cm, with a sharp increase at higher intensities. For instance, fields of 1500 V/cm resulted in free hemoglobin levels of 5.7 g/L, a 71.25‐fold increase compared to the control group (0.07 ± 0.04 g/L).

Mattison et al. [34] investigated the impact of dose and catheter‐tissue contact on PFA‐induced hemolysis in a porcine model using the PulseSelect system (Medtronic). Plasma free hemoglobin (PFH) increased linearly with the number of applications, rising by 0.0003 g/dL per application in the contact group and 0.00056 g/dL in the noncontact group. Noncontact placement resulted in higher hemolysis, highlighting the importance of proper tissue contact. While moderate hemolysis was observed, PFH levels remained below the threshold for severe hemolysis (0.1 g/dL), even after 128 applications, emphasizing the need for optimized catheter positioning and dose management.

Nies et al. [31] examined hemolysis both in vitro and in vivo. In the in vitro experiments, swine blood was exposed to PFA using a pentaspline catheter (Farawave; Boston Scientific) under two conditions: “no‐contact” where the catheter floated freely in the blood, and “in‐contact,” where it was positioned against tissue. Blood samples were collected at baseline and after 2, 4, 8, 12, and 20 applications. Hemolysis occurred in all experiments, but it was significantly higher in the no‐contact condition. After four applications, free hemoglobin levels reached 0.12 ± 0.03 g/dL in the no‐contact group compared to 0.05 ± 0.03 g/dL in the in‐contact group (*p* = 0.008). In vivo experiments with swine undergoing 20–29 PFA applications in the inferior vena cava confirmed similar trends, with a significant post‐ablation increase in free hemoglobin levels.

These insights underscore the importance of PFA catheter design and the need to optimize the PFA waveform to minimize hemolysis and its clinical consequences.

### 3.3 Clinical Implications

The clinical implications of hemolysis following PFA vary significantly depending on the type of catheter used. Differences in catheter design, particularly the extent of tissue contact they enable, and the waveform characteristics contribute to these variations.

The clinical sequelae of hemolysis during PFA are multifaceted:

- Hemoglobinuria and Acute Kidney Injury: Free hemoglobin released into the bloodstream can accumulate in the kidneys, potentially leading to hemoglobinuria and acute tubular damage.
- Thromboembolic Risk: Red blood cell fragments and exposed hemoglobin can activate platelets and the coagulation cascade, increasing the likelihood of thromboembolism.
- Inflammatory Response: Free hemoglobin and other cell breakdown products are potent inducers of inflammation, potentially exacerbating post‐procedural recovery or complications.

The Table 2 summarizes clinical studies examining the incidence of hemolysis, acute kidney injury (AKI), and other hemolysis‐related complications.

**TABLE 2:** Clinical studies investigating hemolysis.

Study name/Author	Hemolysis/Hemolysis‐related complications
MANIFEST‐17K [17]	0.03% Hemolysis renal failure (hospitalization) 0.006% Hemolysis renal failure (no hospitalization)
Popa et al. (2024) [28]	94.3% Hemolysis; 36.4% hemoglobinuria
Venier et al. (2024) [27]	28% patients with haptoglobin < 0.04 g/L 27% hemoglobinuria 3% significant increase in creatinine levels
Stojadinović et al. (2024) [35]	89% major hemolysis (fHb > 500 mg/L)
Kamsani et al. (2024) [36]	Biomarkers for hemolysis increased, but no clinical hemolysis or AKI observed
Jordan et al. (2024) [29]	Hemolysis markers (LDH, bilirubin) elevated in 13.5% of patients; 1% AKI rate

### 3.4 Preventive Strategies

To minimize hemolysis during PFA, several strategies can be employed:

1. Optimizing Number of PFA Application: Studies, including the MANIFEST‐17K registry and others, highlight a direct correlation between the number of PFA applications and hemolysis severity. Reducing the number of energy applications below the thresholds identified (For example, 70 applications in Venier et al., and 74 in Stojadinović et al. for Farapulse PFA system) significantly decreases the risk of hemolysis and its complications [17, 27, 35].
2. Ensuring Proper Catheter‐Tissue Contact: Preclinical studies have shown that a lack of contact between the catheter and tissue increases hemolysis, as more energy is dissipated into the blood [31, 34].
3. Hydration Protocols: Administering adequate hydration during and after the procedure helps mitigate hemolysis‐related complications, such as hemoglobinuria and acute kidney injury (AKI). Studies have shown that patients receiving hydration (e.g., 2 L) post‐PFA had significantly reduced incidences of AKI [17, 27, 28, 32, 35, 36].
4. Energy Delivery Parameters: Adjusting waveform parameters, such as limiting peak voltages or optimizing pulse duration, has been suggested as a means to reduce red blood cell (RBC) electroporation without compromising ablation efficacy [33].
5. Optimizing Electrode Design: Employing electrode shapes that minimize current density hotspots to reduce localized current density on erythrocytes.

These strategies emphasize balancing the therapeutic efficacy of PFA with the reduction of hemolysis to improve patient outcomes and procedural safety.

## 4 Conclusion

Although clinical studies and real‐world data generally support the safety of Pulsed Field Ablation (PFA) for treating arrhythmia, emerging energy‐related complications highlight the need for continued vigilance and refinement of procedural techniques. Gaseous microbubble formation and hemolysis represent key risks directly associated with the electrical field application, distinguishing them from mechanical or procedural complications seen with PFA and other ablation modalities. These concerns highlight the need for a more comprehensive understanding of PFA's biophysical interactions with tissue and blood components, as its effects on different tissues remain incompletely understood. Beyond hemolysis, additional physiological responses have been observed, including smooth muscle spasm, coronary spasms, pronounced vagal reactions, and thermal effects in the esophagus.

Recent safety evaluations have also identified procedural factors that may increase the risk of neurovascular events, as seen in the temporary pause of the Varipulse PFA catheter (Johnson & Johnson MedTech, USA). Excessive ablations, stacking, and non‐PV applications were linked to higher risk, prompting updates to device Instructions for Use (IFU) to improve procedural safety. Similarly, findings from Farapulse PFA system (Boston Scientific Inc., USA) suggest that limiting the number of PFA applications may reduce hemolysis risk, reinforcing the importance of procedural optimization.

Mitigating these complications requires a multifaceted approach, including refinements in the waveforms, improved catheter designs, optimized energy delivery settings, and procedural adaptations to minimize unintended effects.

## Conflicts of Interest

The author declares no conflicts of interest.

## Data Availability

Data sharing not applicable to this article as no datasets were generated or analyzed during the current study.
